# Quantifying and Modeling Birth Order Effects in Autism

**DOI:** 10.1371/journal.pone.0026418

**Published:** 2011-10-19

**Authors:** Tychele Turner, Vasyl Pihur, Aravinda Chakravarti

**Affiliations:** Center for Complex Disease Genomics, McKusick-Nathans Institute of Genetic Medicine, Johns Hopkins University School of Medicine, Baltimore, Maryland, United States of America; The University of Western Australia, Australia

## Abstract

Autism is a complex genetic disorder with multiple etiologies whose molecular genetic basis is not fully understood. Although a number of rare mutations and dosage abnormalities are specific to autism, these explain no more than 10% of all cases. The high heritability of autism and low recurrence risk suggests multifactorial inheritance from numerous loci but other factors also intervene to modulate risk. In this study, we examine the effect of birth rank on disease risk which is not expected for purely hereditary genetic models.

We analyzed the data from three publicly available autism family collections in the USA for potential birth order effects and studied the statistical properties of three tests to show that adequate power to detect these effects exist. We detect statistically significant, yet varying, patterns of birth order effects across these collections. In multiplex families, we identify V-shaped effects where middle births are at high risk; in simplex families, we demonstrate linear effects where risk increases with each additional birth. Moreover, the birth order effect is gender-dependent in the simplex collection. It is currently unknown whether these patterns arise from ascertainment biases or biological factors. Nevertheless, further investigation of parental age-dependent risks yields patterns similar to those observed and could potentially explain part of the increased risk. A search for genes considering these patterns is likely to increase statistical power and uncover novel molecular etiologies.

## Introduction

Autism is a genetic disorder whose molecular basis is incompletely understood. The characterized genetic component of autism, to date, accounts for only <10% of all cases [1]. The most convincing genetic etiologies arise from syndromes which display autism as one part of the phenotype, such as the Fragile(X) [2] and Rett [3], or other single genes with rare mutations in autism, such as those in SHANK3 [4], [5]. Among patients with a diagnosis of autism spectrum disorder (ASD), an excess of maternal 15q11–13 duplications [6] and other chromosomal abnormalities are observed in a small subset of cases. Most patients have no identifiable molecular mutation. However, at the phenotypic level, the increased concordance in identical versus fraternal twins, as well as the increased risk of autism spectrum disorders to siblings of a proband, indicate that autism is highly heritable [7], [8]. Familial patterns of autism clearly show that the disorder is “sex-dependent multifactorial” with numerous genes, likely interacting with environmental and lifestyle factors, increasing risk to family members of a proband and affecting four times as many males as females [9], [10]. The genetic basis for these inherited factors remains to be discovered.

A central feature of most genetic models is that the risk of affection depends on genotype but not position within the family. The latter is rare for a Mendelian disorder with exceptions occurring where disease mutations are strongly dependent on parental age and/or the mutation is dynamic [11]. In contrast, for complex diseases, empirical risks often depend on birth rank. Indeed, for autism, there is some evidence for such a birth order effect for disease severity. In the first study, 16 multiplex families showed a birth order effect where the second affected child tended to have a lower nonverbal IQ [12]. In a follow-up study of 144 multiplex families, the authors found that there was a significant decrease of nonverbal IQ score in the second affected child [13], confirming this effect. A third study of birth order in 106 multiplex families also demonstrated various effects on autism symptom domains across birth ranks [14]. The biological causes of these findings remain unexplored and the generality of birth rank effects in autism has not been systematically investigated.

A birth order effect can arise from both biological (genetic) or demographic (social) causes. Irrespective of etiology, this risk may increase or decrease with birth rank so that the last-borns or first-borns, respectively, are more likely to be affected [15]. In addition, a V-shaped risk distribution in which the middle ranks within a family are either the most or least (inverse V-shaped) likely to be affected has been suggested as a possibility [16].

The simplest demographic cause of an apparent birth order effect is the curtailment of reproduction after the birth of a child with a serious illness. More generally, one might consider social reproduction models where the probability of future births is dependent on disease severity and the number of pre-existing affected children, thus giving rise to patterns where affected offspring are skewed towards later births. A second demographic explanation for the birth order effect can be attributed to intense disease surveillance and curtailment within particular reproductive age-groups so that almost all incident cases occur outside that age-group. For example, in Down syndrome the risk increases with maternal age but in western societies with maternal-age based screening most affected births occur to younger women.

In addition to demographic considerations, there are several well-documented biological causes for birth order effects that act through age. First, the risk of disease may depend on paternal or maternal age and thus increase with increasing birth rank [17]. For example, the risk of an aneuploid zygote, such as trisomy 21, increases significantly with maternal age [18] while some *de novo* gene mutations, such as in achondroplasia, increase with paternal age [19], [20]. Other, more complicated, patterns could arise due to maternal-fetal incompatibility, that is rank- and not age-dependent, such as in Rh-disease, where earlier pregnancies increasingly sensitize the mother towards later pregnancies [21], [22]. Finally, although not completely deduced at this time, epigenetic effects could be one additional factor impacting birth rank effects [23]. All these scenarios, be they demographic or biological, suggest that birth order effects either increase or decrease across birth ranks. Invoking either demographic or biological models for a V-shaped birth rank distribution is admittedly difficult but statistically feasible. Nevertheless, it is quite likely that these arise where the causes are heterogeneous with some families showing an increasing and others a decreasing birth effect trend.

To the best of our knowledge, a comprehensive formal assessment of overall birth order effect in autism, at least in the families now being extensively used for genetic investigations, has not been attempted. Consequently, in this study, we explore whether such birth rank trends exist in the three major and widely-available US autism family collections, namely, AGRE (Autism Genome Resource Exchange) [24], NIMH (National Institute of Mental Health: https://www.nimhgenetics.org/), and SSC (Simons Simplex Collection) [25]. Previous studies in autism have used a two sample paired t-test to assess autism severity differences between siblings of early and late births. In this manuscript, we propose three nonparametric statistical tests that *directly* examine deviations from the expected uniformity in the distribution of affected offspring across birth ranks. The proposed testing procedures differ in their efficiency depending on the shape of the birth order effect as shown by the results of our systematic simulation studies. Having access to large autism collections with hundreds of families allows a test for birth order effect in both simplex and multiplex families. We demonstrate that these effects are statistically significant and that different collections vary in the patterns they manifest. Consequently, these effects impact the search for autism genes.

## Materials and Methods

### Autism Family Collections

This study was conducted under ethics approval by the Johns Hopkins University School of Medicine Institutional Review Board (IRB) and was deemed to be exempt from full IRB review under exemption category 4. The families examined in this study were ascertained by the Autism Genome Resource Exchange (called the AGRE collection) [24], the National Institute of Mental Health (NIMH) (https://www.nimhgenetics.org/), and the Simons Foundation (Simons Simplex Collection (SSC)) [25]. All data were collected by AGRE, NIMH, and SSC under their respective IRBs. The AGRE and NIMH collections are primarily composed of multiplex families whereas the SSC is a simplex family collection. All samples were ascertained within the United States; selected features of the cases and families used in this study are summarized in Table 1. The main criterion for including families in the birth order study was choosing two-parent families with a minimum of two offspring with known birth dates. Additionally, we excluded families with discordant twins and counted concordant twins as one individual.

**Table 1:** Summary statistics of autism collections.

Family Collection	# families	# children	# affected	sibship size	# affected sibs
AGRE	346	1,234	744	3–9	2–5
NIMH	396	1,393	840	3–10	2–5
AGRE + NIMH	485	1,716	1,030	3–10	2–5
SSC	1,119	2,392	1,119	2–6	1

The two multiplex collections used in this study were the NIMH and AGRE collections. For the most part, both sets consist of families with at least two offspring affected with an autism spectrum disorder. The NIMH data comes from the NIMH distribution 6.0 pedigree files while the AGRE data was obtained from the AGRE January 2010 pedigree files. For AGRE, year of birth data was obtained from Vlad Kustanovich at AGRE (personal communication, 2010). Across all families, we counted all those with a diagnosis of autism or ASD as affected. In the AGRE collection, 346 families met these criteria and contained 744 individuals with autism (60%) among a total of 1,234 children. In the NIMH collection, 396 families met these criteria and contained 840 individuals with autism (60%) among a total of 1,393 children. These two collections have families in common so that we combined the data sources to obtain a single non-duplicated dataset of 485 families containing 1,030 individuals with autism (60%) among a total of 1,716 children. The Simons Simplex Collection (SSC) consists of simplex families with at least two offspring only one of whom had a diagnosis of ASD. We obtained the data from the SSC v.7.1 data release to identify 1,119 individuals with autism (47%) among a total of 2,392 children. The high frequency of autism in all families show the ascertainment bias induced in these collections.

### Statistical Methods

We extended and generalized three different tests for the birth order effect and assessed their statistical power by computer simulations under different, but biologically relevant, types of disease risk profiles. A software package implementing these tests is freely available from the authors. A brief description of each test follows.

The rank-sum test [15] is perhaps the most intuitive non-parametric test for testing a birth order effect. In a sibship of size two or greater let 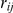 denote the birth position of the 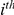 child in the 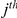 family. We compare the sum of 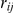
's for affected individuals with what would be expected in the absence of a birth order effect, i.e. a discrete uniform distribution on [1,N] of being affected at any birth position given the sibship size N>2. The test statistic is defined as:



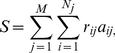
(1)where 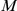 is the number of families, 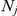 is the number of siblings in family 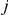 and,



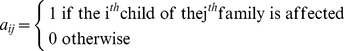
(2)To test the null hypotheses of no birth order effect, we perform a permutation test to obtain the distribution of 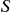 under the null hypothesis. To accomplish this, for the same number of families 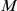 and the same number of children in each family 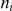, we randomly permute the disease status 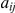 within each family 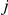. This general scheme preserves the overall structure and dependence in our observed data, including the number of affected offspring in each family, while generating a possible realization of the data under the null hypothesis. For each permuted null outcome, we compute the test statistics 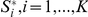 and the two-sided p-value as:



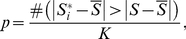
(3)Where 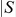 is the absolute value of 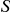, 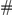 denotes the number of times, 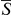 is the mean of 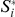
′s and K is the number of permutations.

Although the above test may be useful for linear changes in risk, it is inefficient whenever the risk distribution across births is V-shaped [16]. To remedy this situation, we consider an alternative ranking scheme where birth ranks are also weighted in a V-shaped manner (Table S1). The test statistics, permutation procedure and hypothesis testing are exactly the same as above (equations 1–3). The advantage of the new ranking is that it is capable of detecting complicated patterns of birth order effect, for example, when the probability of being affected is large for both early and late born offsprings. We call this the ‘inverse rank-sum test.’

The third test of the birth order is a direct comparison of the observed and expected numbers of affecteds at each birth rank using a χ^2^-type test [26] that 

uses the actual counts of affected offspring at each birth position and not the ranks (Table S2). The test statistic has the familiar form of



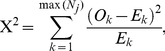
(4)and yet the distribution under the null hypothesis is not a central χ^2^ distribution because the observations are not independent. The observed counts for the 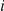
th birth rank are simply calculated as the total counts across appropriate sibship sizes, i.e.,
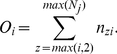
(5)The expected counts for the 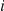
th birth rank are computed under the assumption of uniform distribution of affected siblings within each sibship size. Thus, 
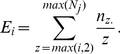
(6)Analogous to the first two tests, we resort to a permutation technique to assess statistical significance and to obtain a p-value.

## Results

Before analysis of the real data, we first assessed statistical power and the optimal behavior of the three tests under a variety of biological models of birth order effect. We performed extensive Monte-Carlo simulations to easily accommodate a variety of risk models. In the first set of simulations, we assumed five simple models where disease risk was (a) independent of birth rank, (b) linearly dependent on birth rank (we tested only an increasing risk since the decreasing model is statistically equivalent), (c) V-shaped birth order effect; (d) and (e) were mixtures of linear but opposite birth rank trends in 9∶1 (increasing/decreasing) and 3∶7 proportions. To generate family structures, we used a Poisson distribution for sibship sizes with mean λ = 2.39 (the US average value). The probability of being affected at each birth rank *z* was computed as a linear function of birth ranks as:
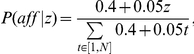



where N is the sibship size. Both intercept and slope were selected such that the increased risk was substantial but not exceedingly large.

We estimated statistical power for 500 and 1,000 families under the five linear models. These results are shown in Table 2. All three statistical tests are well-behaved in that the nominal Type I error is below 5% under the null hypothesis. As expected, the power increases with the number of families analyzed and can be considerable even for 500 families. Among the three tests, the rank-sum statistic has the largest power for detecting linear trends (including a mixture of linear effects), while the inverse rank-sum test is best for V-shaped effects, as expected. Interestingly, the χ^2^–type test proves to be a good compromise between the two other tests and is capable of detecting both types of nonuniform birth effects in the data. Its robust performance makes it the test of choice for the birth order effect, although it is less powerful than at least one of the other tests when the risk effects are either linear or V-shaped. In contrast, one has to keep in mind that both the rank-sum and inverse rank-sum test are likely to miss more complicated patterns of birth order effects.

**Table 2:** Linear trend power calculations for three birth order statistical tests.

N	Test	Birth order model				
		Uniform	Linear	V-shaped	Mixed linear (p_0_ = 0.9)	Mixed linear (p_0_ = 0.3)
500	Rank-sum	0.05	0.74	0.06	0.55	0.14
	Inverse rank-sum	0.04	0.07	0.28	0.07	0.06
	χ^2^-type	0.06	0.38	0.03	0.25	0.03
1000	Rank-sum	0.03	0.97	0.05	0.82	0.29
	Inverse rank-sum	0.01	0.06	0.45	0.07	0.02
	χ^2^-type	0.01	0.68	0.10	0.43	0.10

Although the previous power calculations are encouraging, we wanted to model risk using known genetic mechanisms. In other words, we know of many genetic mechanisms that vary risk by age as opposed to birth order. Thus, we wanted to assess how age-dependent risks would manifest themselves as birth rank effects. A commonly accepted biological risk model is logistic where risk of disease is age-dependent and the precise dependence is modeled by three parameters: β (intrinsic increase/decrease rate), *b* (the maximum risk) and *a* (age-factor whereby an individual reaches half the maximum risk):
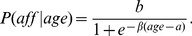
(7)


**Figure 1:**
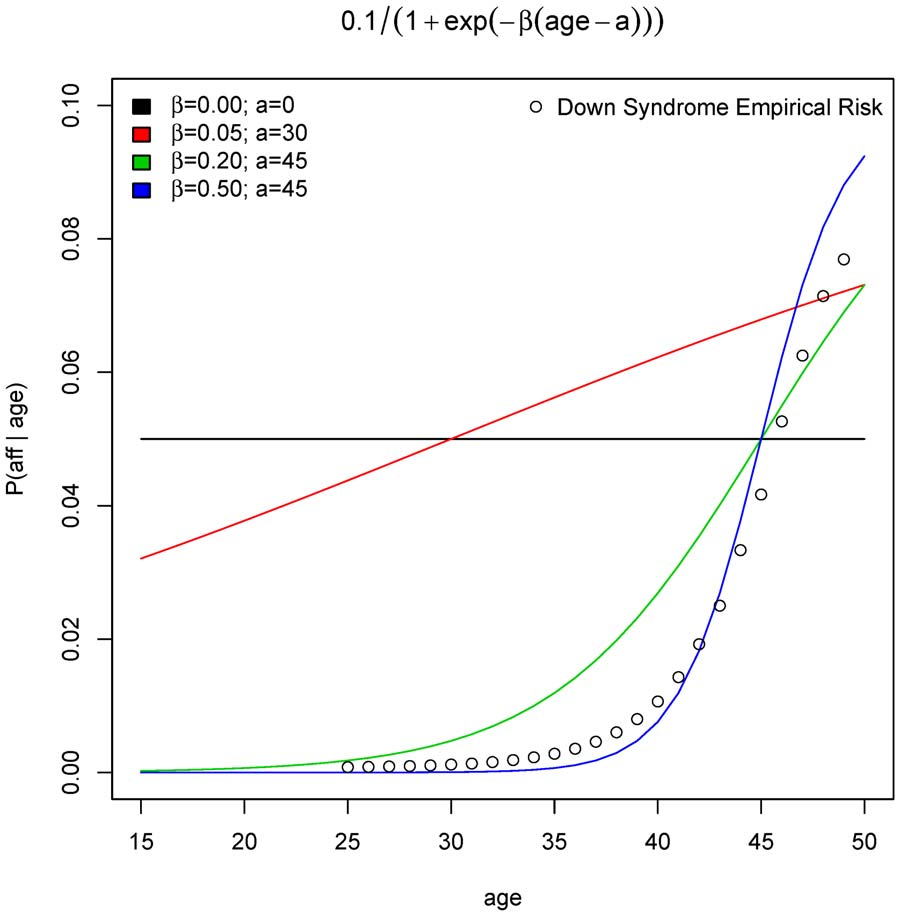
Logistic models for disease risk as a function of parental age; *β* is the rate of increase of logistic risk and *a* is the age in years at which risk is 50%.

We considered four sets of parameters to model three classes of risk, namely, where disease risk was (a) independent of age, (b) linearly increasing with age (we tested only an increasing risk since the decreasing model is statistically equivalent), (c) logistic increase in risk with lower and higher rates of increase at later ages. These risk profiles are shown in Figure 1 with *b* assumed to be 0.1 for all cases. Empirically observed risk rates for Down Syndrome that shows a maternal-age effect are also plotted in open circles providing a reasonable benchmark for the four models. To generate family structures, we again used a Poisson distribution for sibship sizes with mean λ = 2.39. Parameter selection for the four models was motivated by presenting reasonable yet somewhat different risk patterns. In the no-birth effect model both β and *a* are set to 0; for the linear model, β = 0.05 and *a* = 30, and for the two logistic curves, *a* = 45 is selected as the age at which the risk reaches half of its maximum *b* at two different rates (β = 0.2 and β = 0.5). The model with β = 0.5 and *a = 45* is clearly the one closest to the observed risk pattern in Down Syndrome and is of primary interest as a potential ‘model’ for the autism age-dependent risk profile.

**Figure 2:**
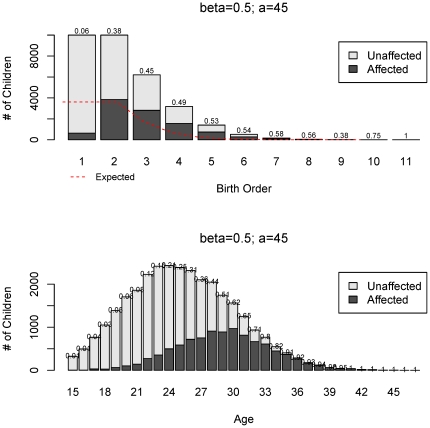
Example of effect of age-dependent logistic risk on birth order effect. For the most extreme age-dependent risk increase in Figure 1 (*β* = 0.5; *a* = 45) we show the distribution of offspring, both affected and unaffected, by (a) birth rank, and (b) parental age, by simulations of 10,000 nuclear families with Poisson distribution of sibship size with mean 2.39. At the top of each bar the fraction of affected offspring at that birth rank is shown.

**Figure 3:**
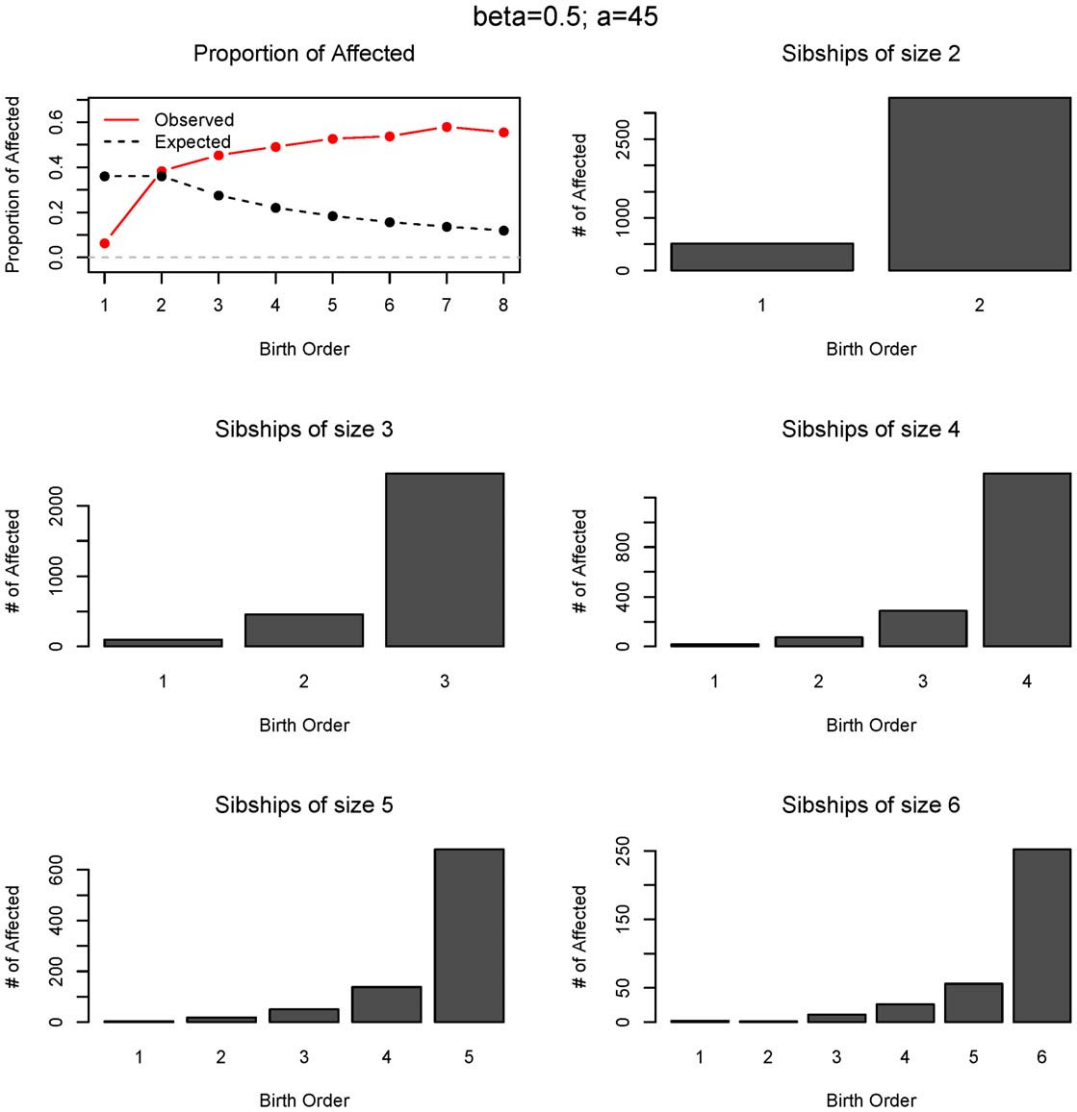
Statistical distribution of birth order effect of the data in **Figure 2**. Proportion of observed and expected numbers of affected offspring at each birth rank and the numbers of affected offspring for each sibship size and birth order is shown.

**Figure 4:**
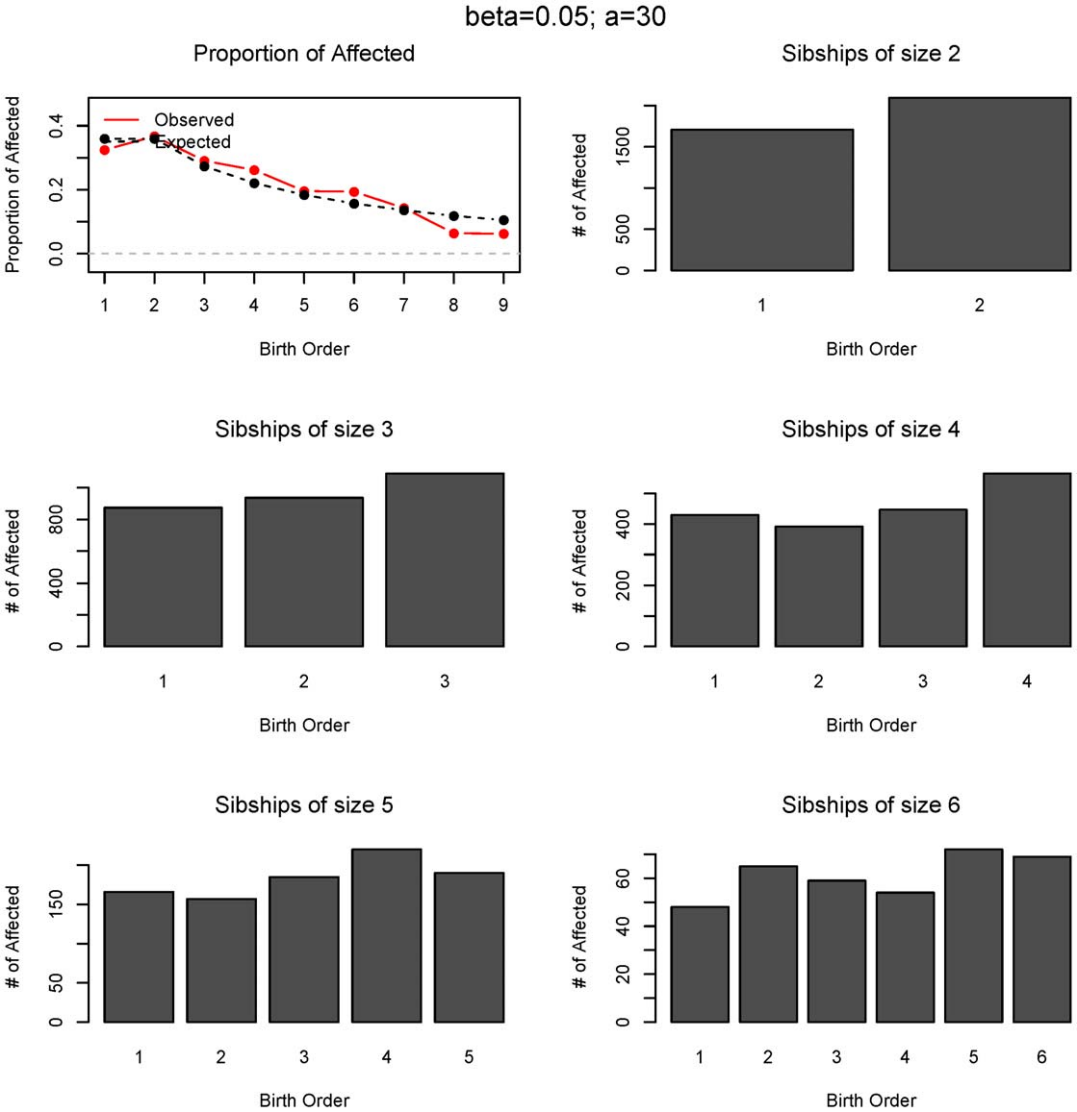
Statistical distribution of birth order effect for the linear model (*β* = 0.05; *a* = 30). Proportion of observed and expected numbers of affected offspring at each birth rank and the numbers of affected offspring for each sibship size and birth order is shown. Linear risk increase in parental age translates into an approximately linear risk increase in birth ranks.

To examine the nature of birth rank effects recovered we simulated 10,000 families with logistic risks and the parameters β = 0.5 and *a = 45*. The proportion of affected offspring by birth rank and the distributions of parental age for both affected and unaffected offspring are shown in Figure 2. The shift to the right in the two age distributions is very apparent and is also reflected in the birth ranks, especially in the deficiency of the first-born affected children. Figure 3 demonstrates the exponential increase in risk for all sibship sizes in *birth rank*, measured as proportion of affected at each rank, which directly corresponds to the underlying increase in risk by parental age. The expected and observed proportions of affected offsprings by birth rank differ substantially (top left) and the χ^2^-type test is highly significant (p<0.001). In Figure 4, we examine a linear risk model as a function of parent's age that translates approximately into a linear risk in birth ranks. This model corresponds to β = 0.05 and *a = 30* and is also plotted in red in Figure 1. The χ^2^-type test for the birth order effect in these 10,000 families is also highly significant (p<0.001) although the departure of observed to expected proportions is not as substantial as in the previous logistic example. Nevertheless, we still observe an increase in risk at each sibship size and the overall test confirms this effect. To estimate the statistical power for these logistic models we again resort to Monte-Carlo simulations. The estimated power for sample sizes 150, 500 and 1,000 families based on 100 simulations is shown in Table 3. Clearly, the rank-sum test has the largest power in all scenarios, followed by the χ^2^-type test. As expected, the inverse rank-sum test has the least power for monotonic patterns of risk increase and should not be used in such cases.

**Table 3:** Logistic model power calculations for three birth order statistical tests.

N	Test	Birth order model			
		β = 0; a = 0	β = 0.05; a = 30	β = 0.2; a = 45	β = 0.5; a = 45
150	Rank-sum	0.06	0.23	1.00	1.00
	Inverse rank-sum	0.06	0.07	0.46	1.00
	χ^2^-type	0.03	0.14	1.00	1.00
500	Rank-sum	0.03	0.64	1.00	1.00
	Inverse rank-sum	0.01	0.04	0.91	1.00
	χ^2^-type	0.03	0.33	1.00	1.00
1000	Rank-sum	0.04	0.87	1.00	1.00
	Inverse rank-sum	0.04	0.03	1.00	1.00
	χ^2^-type	0.05	0.54	1.00	1.00

Finally, we utilized all three statistical tests and 10,000 permutations each to assess birth order effects in the multiplex AGRE and NIMH data sets and the simplex SSC data set; these results are presented in Table 4. Three trends are evident from these analyses. First, all three autism collections show statistically significant evidence of birth order effects in at least one test. Second, the χ^2^–type test was significant in all three autism collections. Third, the results from the multiplex collections are more significant than those from the simplex families.

**Table 4:** Results of three birth order tests in the autism collections.

Family Collection	Rank-sum test	Inverse rank-sum test	χ^2^-type test
AGRE	0.786	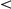 10^−4^	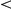 10^−4^
NIMH	0.500	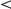 10^−4^	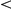 10^−4^
SSC	0.013	0.066	0.017

**Figure 5:**
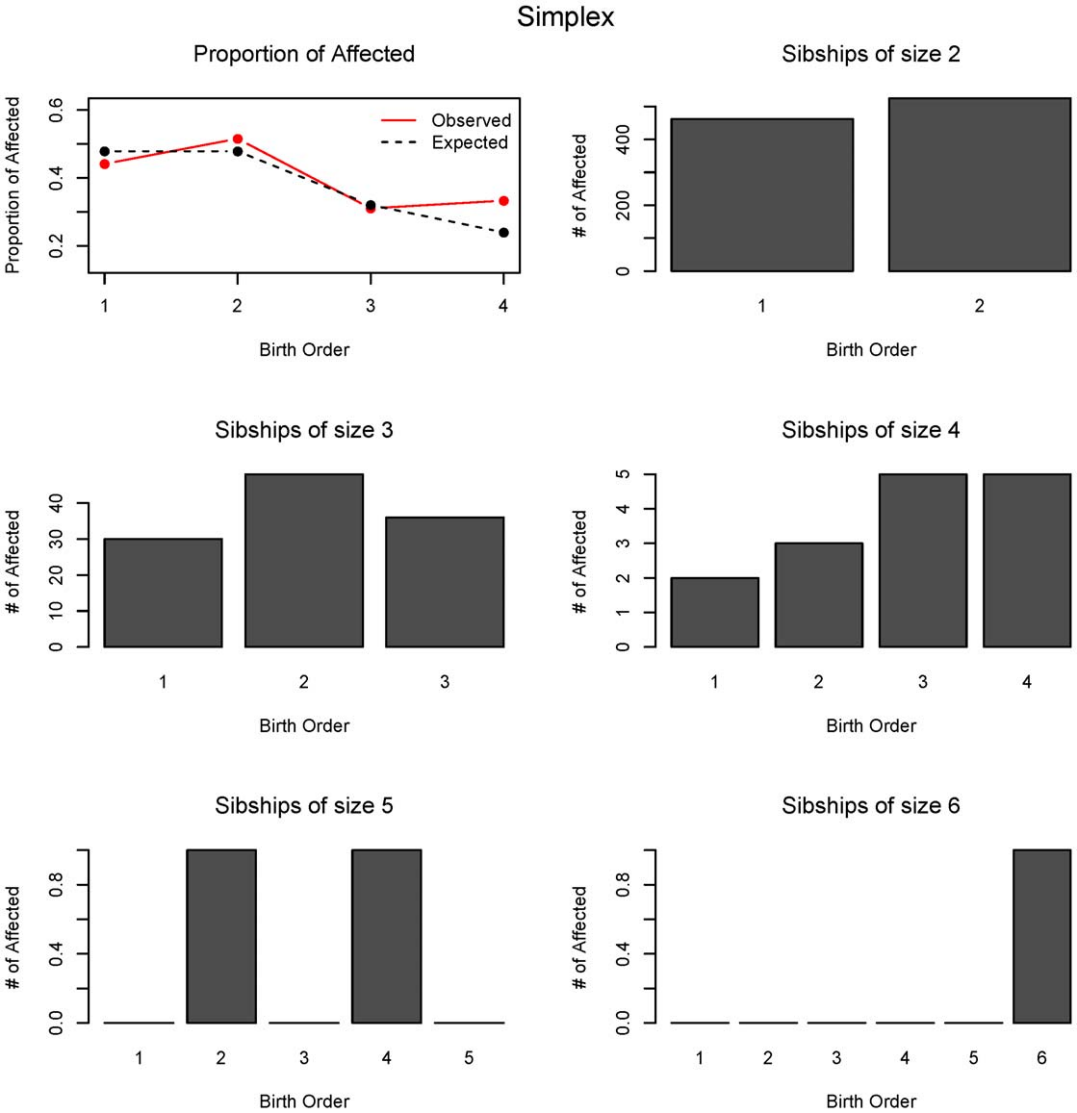
Proportion of observed and expected numbers of affected offspring at each birth rank for simplex families. The data for the numbers of affected offspring for each sibship size and birth order is shown.

**Figure 6:**
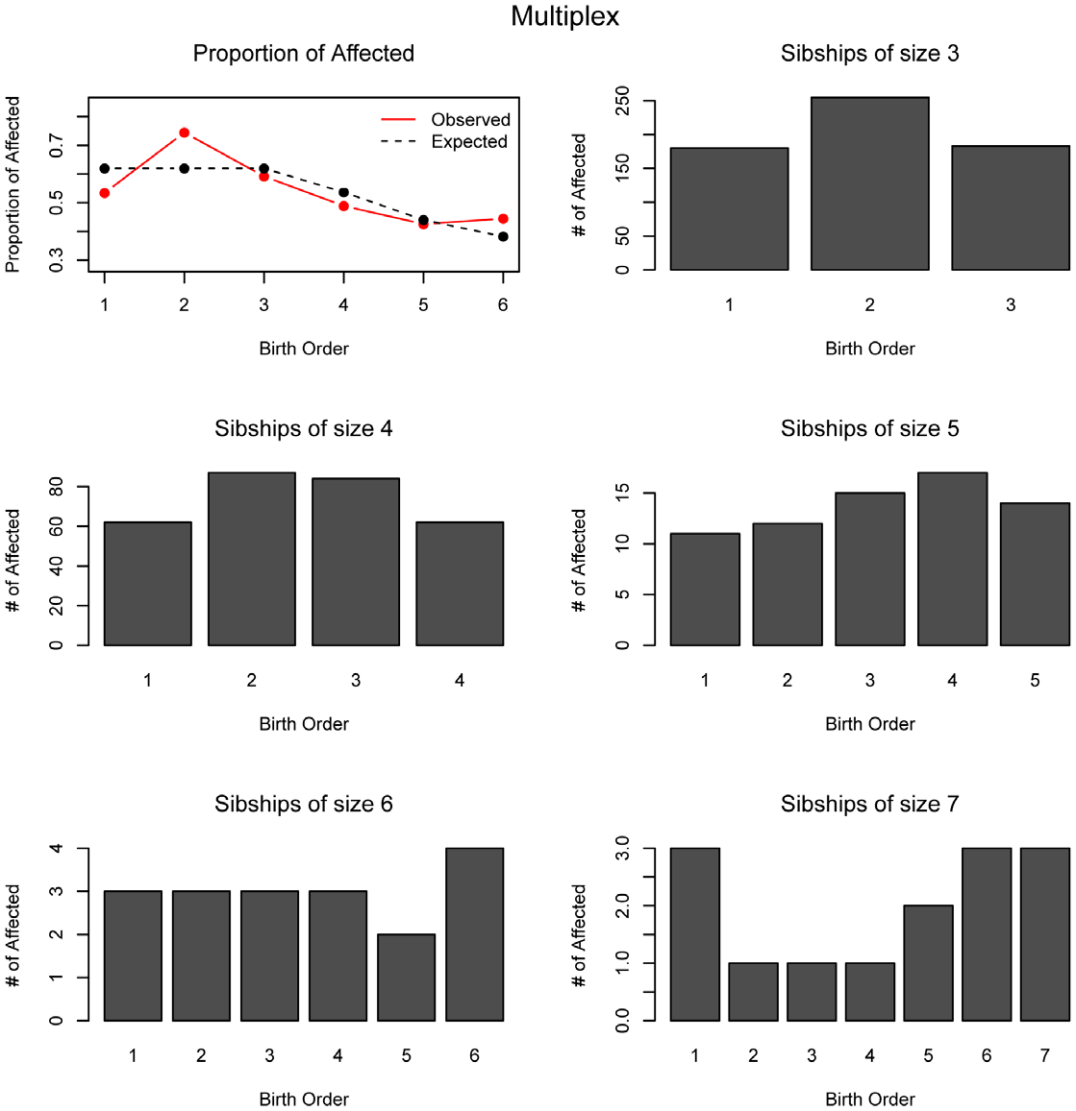
Proportion of observed and expected affected offspring at each birth rank for multiplex families. The data for the numbers of affected offspring for each sibship size and birth order is shown.

Based on the results of power analysis and assumed birth order effects, we can infer that the multiplex collections are not significant for the linear effect (rank-sum test) but very significant for the V-shaped effect (inverse rank-sum test), whereas the SSC showed an opposite pattern. In the combination dataset (AGRE + NIMH), shown in Table 5, we obtained insignificant results for the linear trend but significant results for the V-shaped effect. Thus overall, in both sets of families, the multiplex families are different in their risk profiles across births than the simplex families. Furthermore, in simplex families there is an excess of the affecteds at rank two which is the main driver for the observed birth order effect (Figure 5). On the other hand, in multiplex families the excess at rank 2 is followed by below expected counts in the following birth ranks, contributing to the inverse V-shape effect (Figure 6).

**Table 5:** Results of three birth order effect tests in the autism collections by gender.

Family Collection	# of affected	# of families	rank-sum test	inverse rank-sum test	χ^2^-type test
AGRE + NIMH	1,030	485	0.382	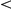 10^−4^	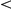 10^−4^
AGRE + NIMH (affected females only)	58	29	0.354	0.0096	0.0409
AGRE + NIMH (affected males only)	658	317	0.959	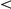 10^−4^	0.014
SSC	1,119	1,119	0.013	0.066	0.017
SSC (affected females only)	150	150	0.410	0.460	0.819
SSC (affected males only)	969	969	0.004	0.076	0.005

Due to large gender differences in autism prevalence, gender specific analyses in each of the collections were performed. Intriguingly, as reported in Table 5, gender specific birth order results are identified only within the SSC. For the AGRE + NIMH families both female-only and male-only families show significance for a V-shaped birth effect with a greater significance in males likely due to their larger sample size. Whether the genetic effect is larger in female-only families cannot be assessed with the sample sizes available (n =  58 affected females in 29 families). This multiplex effect shows a greater number of affecteds at the middle ranks peaking at birth rank 2 (Figure 6). On the other hand, for the SSC, affected females do not exhibit any birth order effect, whereas, affected males have a very significant linear increasing birth order effect (Figure 5, Figure S1). The effect is greater in simplex male-only affected families than it is in the overall simplex families. The absence of effect in simplex female-only affected families supports the idea that females, who are less commonly affected, show a more characteristic genetic etiology. If birth order effects are similar to our simulated example, there is some evidence that we should have had enough detection power if it were true.

To examine both collections for parental age we also studied the parental ages for all individuals for whom this information was available. Overall, the distribution of affected children and unaffected children across parental ages did not differ (Figure S2) but the proportion of affected children of all the children showed trends very similar to the birth order effects (Figure S3).

## Discussion

In this study, we analyzed three autism datasets (AGRE, NIMH, SSC) for familial birth order effects in multiplex and simplex families. To accomplish this we generalized and implemented three different statistical tests for birth order effects and carried out a series of simulations studies to assess their performance under different birth order patterns. We demonstrated the proposed rank-sum, inverse rank-sum and the χ^2^ –type tests to be best at detecting linear, V-shape, and general types of patterns, respectively. The χ^2^ –type test proved to be the most robust test, capable of identifying arbitrary deviations from the expected distribution, although slightly less efficient when the true risk profiles are either linear or V-shaped.

Application of each test to the simplex collection identified significant linear birth order effects confirmed by both the rank-sum and the χ^2^ –type tests. Looking at the results visually confirms an increasing linear effect that peaks around rank 2 (Figure 5). A demographic cause of this effect could arise from curtailment after the affected child is born. This is unlikely because a family size of 2 is common in the USA. More intriguing biological mechanisms include parental age effects, maternal-fetal incompatibility, and potential epigenetic factors. Parental age effects could arise as paternal or maternal. Well documented examples are maternal age effects in Down Syndrome from aneuploidy [18], [27] and paternal age effects in Achondroplasia from recurrent male mutation [19], [20]. Maternal age effects arise from non-disjunction whereas paternal age effects arise from point mutations due to increased numbers of replication in the male germline [28]. Considerations of these effects guide researchers to examine *de novo* mutations (both copy number and substitutions) in individuals with autism. Maternal-fetal incompatibility such as in Rh-disease are difficult to detect with current genomic studies: with increased risk to subsequent births this is a potential consideration for autism etiology. This type of effect should be carefully studied through the development of novel genetic methods. Lastly, epigenetic changes across pregnancies may exist but remain an enigma.

In the multiplex families, the inverse-rank sum test and the χ^2^ –type test were both significant and indicated an inverse V-shape birth order effect in which the middle ranks are more commonly affected as can be seen in Figure 6. They are highest at rank two and decrease until birth rank 5 in which the number of sibships of size 5 are so small that the effect is no longer observed. This V-shaped effect is most likely a result of a heterogenous collection of families with differing birth order effects. A major factor to consider is ascertainment bias when selecting large multiplex families. This bias results because families are not chosen at random but likely with probability proportional to the number of affected children. The simplex collection was not randomly chosen either and families had to meet very stringent criteria to be added to the collection and required an unaffected sibling. Whereas the SSC commonly excluded families with other relatives who also had autism, multiplex families were collected with the purpose of having multiple affected individuals.

This study shows birth order effects do exist in autism and that some underlying biological cause may account for these effects. The *de novo* mutation associated with parental age hypothesis has been put forth in the recent past in particular for simplex families. [29] Certainly, this study adds weight to this hypothesis in that simplex family effects are linear and increasing. In addition, multiplex families do appear to be highly heterogenous and may be a result of *de novo* parental age effects in combination with genetic segregation patterns. One should not immediately discount other possibilities such as ascertainment bias or sibship curtailment. In addition, with the mysterious nature of autism causation it may be time to consider newer models and possibilities such as maternal-fetal incompatibility and/or epigenetic changes. Perhaps this can lead to specific studies of maternal genotype and fetal environment.

## Supporting Information

Figure S1 **The expected and observed numbers of affected males and females by birth rank in simplex and multiplex autism families.**
(TIFF)

Figure S2 **Distribution of parental age at birth of affected (red) and unaffected (black) offspring in simplex and multiplex autism families.**
(TIFF)

Figure S3 **Proportion of affected children of all children at each parental age.**
(TIFF)

Table S1 **The inverse ranking scores used for the inverse rank-sum test.** Offspring that are either early born or late born are given larger linear ranks as compared to the middle born children.
(DOC)

Table S2 **Observed number of affected individuals for each sibship size and birth rank.**
(DOC)
