# Supplementary figures and images for: Quantifying and Modeling Birth Order Effects in Autism
Source: PLoS One. 2011 Oct 19;6(10):e26418. doi: 10.1371/journal.pone.0026418 (PMC3198479; doi:10.1371/journal.pone.0026418)

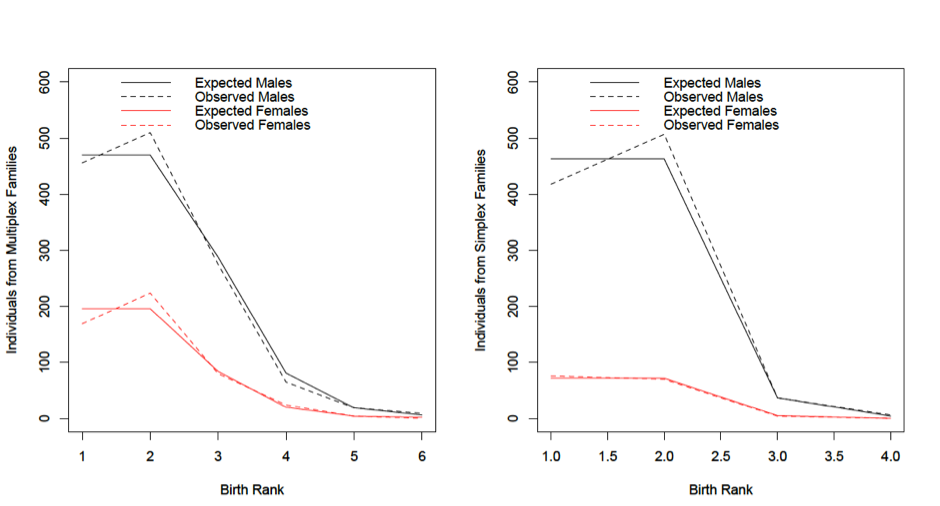

Supplement: Figure S1 — The expected and observed numbers of affected males and females by birth rank in simplex and multiplex autism families. (TIFF) [file pone.0026418.s001.tiff]

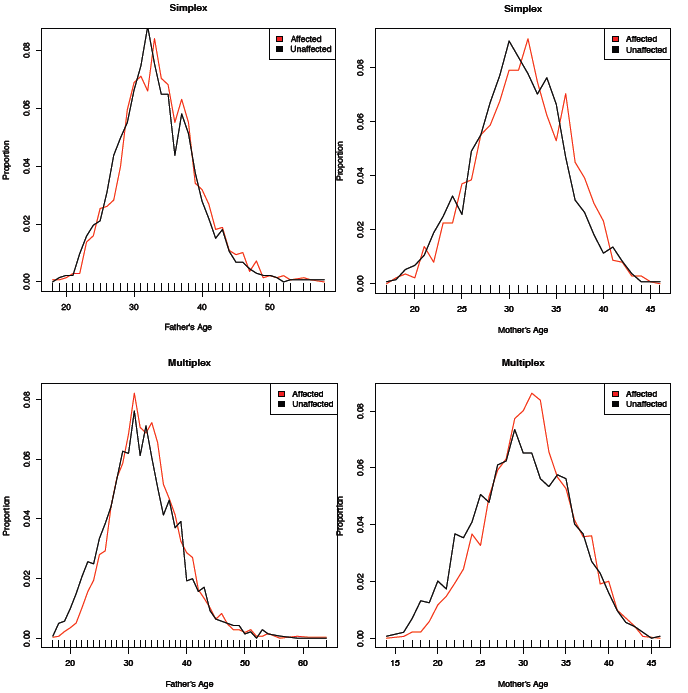

Supplement: Figure S2 — Distribution of parental age at birth of affected (red) and unaffected (black) offspring in simplex and multiplex autism families. (TIFF) [file pone.0026418.s002.tiff]

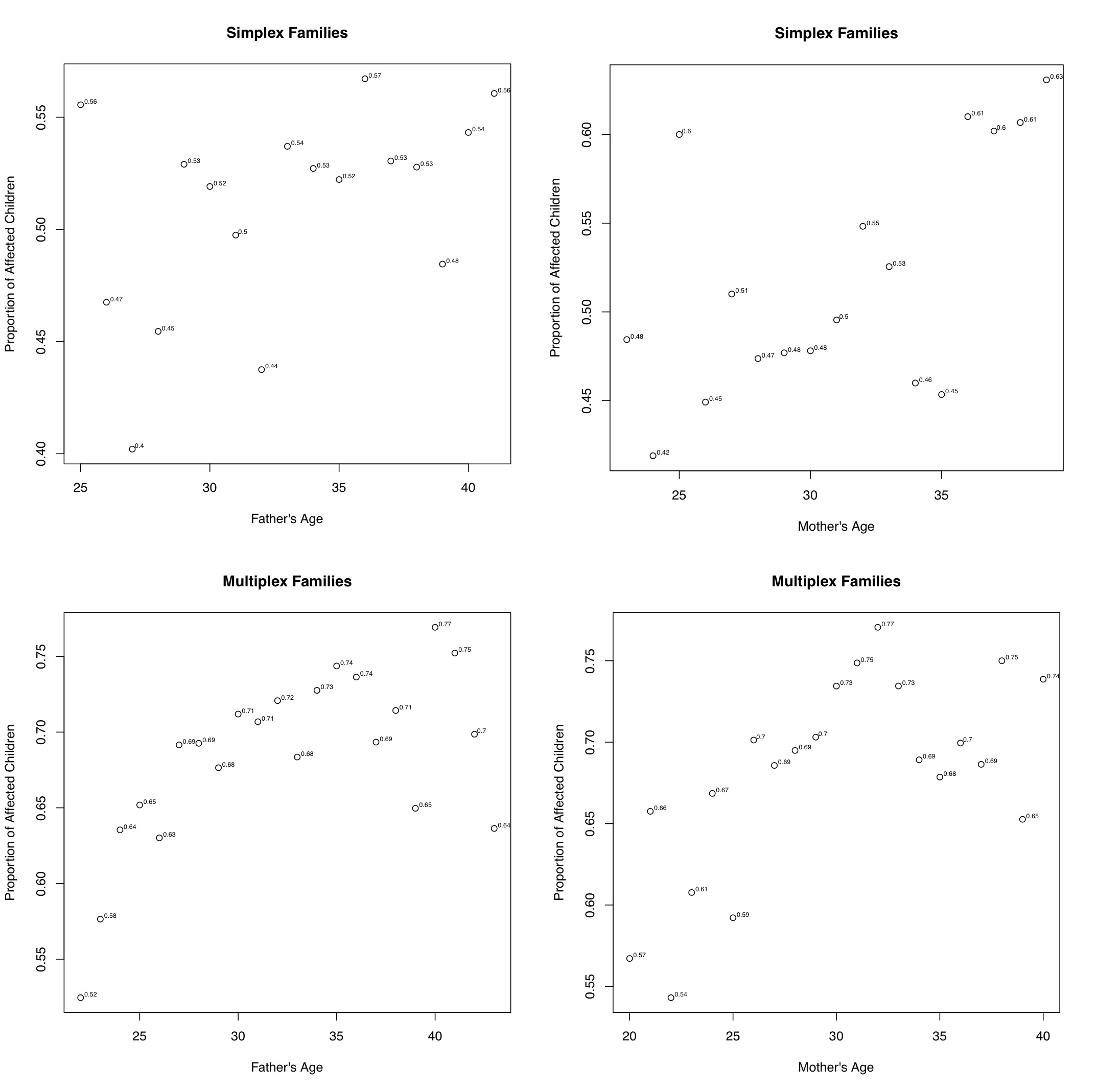

Supplement: Figure S3 — Proportion of affected children of all children at each parental age. (TIFF) [file pone.0026418.s003.tiff]
