# Supplementary material for: Quantifying and Modeling Birth Order Effects in Autism
Source: PLoS One. 2011 Oct 19;6(10):e26418. doi: 10.1371/journal.pone.0026418 (PMC3198479; doi:10.1371/journal.pone.0026418)
Supplement: Table S1 — The inverse ranking scores used for the inverse rank-sum test. Offspring that are either early born or late born are given larger linear ranks as compared to the middle born children. (DOC) [file pone.0026418.s004.doc]

*Table S1: Inverse ranking scores used for the inverse rank-sum test.*

| *Sibship size (N_j_)* | *Birth Rank* | | | | |
| --- | --- | --- | --- | --- | --- |
|  | *1* | *2* | *3* | *4* | *5* |
| 2 | 1 | 1 | 0 | 0 | 0 |
| 3 | 2 | 1 | 2 | 0 | 0 |
| 4 | 2 | 1 | 1 | 2 | 0 |
| 5 | 3 | 2 | 1 | 2 | 3 |
