# Supplementary material for: Quantifying and Modeling Birth Order Effects in Autism
Source: PLoS One. 2011 Oct 19;6(10):e26418. doi: 10.1371/journal.pone.0026418 (PMC3198479; doi:10.1371/journal.pone.0026418)
Supplement: Table S2 — Observed number of affected individuals for each sibship size and birth rank. (DOC) [file pone.0026418.s005.doc]

*Table S2: Observed number of affected individuals for each sibship size and birth rank.*

| Sibship size (*N_j_*) | Birth Rank | | | | | Total |
| --- | --- | --- | --- | --- | --- | --- |
|  | 1 | 2 | 3 | 4 | 5 |  |
| 2 | *n_21_* | *n_22_* | 0 | 0 | 0 | *n_2._* |
| 3 | *n_31_* | *n_32_* | *n_33_* | 0 | 0 | *n_3._* |
| 4 | *n_41_* | *n_42_* | *n_43_* | *n_44_* | 0 | *n_4._* |
| 5 | *n_51_* | *n_52_* | *n_53_* | *n_54_* | *n_55_* | *n_5_,* |
| Observed | *O_1_* | *O_2_* | *O_3_* | *O_4_* | *O_5_* | *n* |
